# Trends over 50 years with liberal abortion laws in the Nordic countries

**DOI:** 10.1371/journal.pone.0305701

**Published:** 2024-07-10

**Authors:** Finn Egil Skjeldestad, Mika Gissler, Reynir Tómas Geirsson, Anna Heino, Hildur Björk Sigbjörnsdottir, Rupali Akerkar, Kristina Gemzell-Danielsson, Oskari Heikinheimo, Mette Løkeland

**Affiliations:** 1 Department of Community Medicine, Research Group Epidemiology of Chronic Diseases, Faculty of Health Sciences, UiT The Arctic University of Norway, Tromsø, Norway; 2 Department of Knowledge Brokers, THL, Finnish Institute for Health and Welfare, Helsinki, Finland; 3 Department of Molecular Medicine and Surgery, Region Stockholm, Academic Primary Health Care Centre, Stockholm, Karolinska Institutet, Stockholm, Sweden; 4 University Department of Obstetrics and Gynecology, Women’s Clinic, Landspitali University Hospital, Reykjavik, Iceland; 5 Faculty of Medicine, University of Iceland, Reykjavik, Iceland; 6 Health Information Section, Directorate of Health, Reykjavik, Iceland; 7 Devision of Health Data and Digitalization, Norwegian Institute of Public Health, Bergen, Norway; 8 Division of Obstetrics and Gynecology, Department of Women’s and Children’s Health, Karolinska Institute, and Karolinska University Hospital, Stockholm, Sweden; 9 Department of Obstetrics and Gynecology, University of Helsinki and Helsinki University Hospital, Helsinki, Finland; 10 Department of Obstetrics and Gynecology, Haukeland University Hospital, Bergen, Norway; United Arab Emirates University, UNITED ARAB EMIRATES

## Abstract

**Background:**

During the 1970s the Nordic countries liberalized their abortion laws.

**Objective:**

We assessed epidemiological trends for induced abortion on all Nordic countries, considered legal similarities and diversities, effects of new medical innovations and changes in practical and legal provisions during the subsequent years.

**Methods:**

New legislation strengthened surveillance of induced abortion in all countries and mandated hospitals that performed abortions to report to national abortion registers. Published data from the Nordic abortion registers were considered and new comparative analyses done. The data cover complete national populations.

**Results and conclusions:**

After an increase in abortion rates during the first years following liberalization, the general abortion rates stabilized and even decreased in all Nordic countries, especially for women under 25 years. From the mid-1980s higher awareness about pregnancy termination led women to present at an earlier gestational age, which was accelerated by the introduction of medical abortion some years later. Most terminations (80–86%) are now done before the 9^th^ gestational week in all countries, primarily by medical rather than surgical means. Introduction of routine ultrasound screening in pregnancy during the late 1980s, increased the number of 2^nd^ trimester abortions on fetal anomaly indications without an overall increase in the proportion of 2^nd^ relative to 1^st^ trimester abortions. Further refinement of ultrasound screening and non-invasive prenatal diagnostic methods led to a slight increase in the proportion of early 2^nd^ trimester abortions after the year 2000. Country-specific differences in abortion rates have remained stable over the 50 years of liberalized abortion laws.

## Introduction

Over the past 50 years there has with some exceptions been a worldwide trend to liberalize abortion laws. The quality and safety of abortion care has improved [1, 2].

In the Nordic countries demands for abortion reforms and for removing the issue from the penal code were raised alongside a public debate on the need for sex education and birth control during the late 1920s and 1930s. Below we describe for each Nordic country how views concerning abortion care gradually changed from restrictive to more liberal, based on predefined indications, and finally to abortion on request.

In Denmark, abortion was first allowed in 1939 if the pregnancy was considered as seriously endangering the health and life of the mother, if there was an elevated risk for birth defects or if the conception was a result of rape [3]. Only a low proportion of the applications were approved until the 1960s. Indications were widened in 1970 to allow induced abortions for women under the age of 18 who were believed to be at risk of becoming "unqualified mothers”. Also, women over the age of 38 were given easier access to abortion. In 1973 the Danish parliament passed a law giving women a right to abortion on request, provided the procedure was done during the first 12 weeks of pregnancy. For women requesting an abortion after the 12^th^ gestational week, the application had to be evaluated by a regional board of three members (gynecologist, social worker, psychiatrist) according to specific circumstances prespecified in the law [3].

The first Finnish legislation dates from 1950, but legal abortion was available only for medical, eugenic and sociomedical indications. Finland legalized abortion on several social indications in 1970 allowing abortion up to 16 gestational weeks if approved by one or two physicians [4]. The gestational age limit was lowered to 12 weeks in 1979 [4]. In 1985 the upper gestational limit was increased from 20 to 24 weeks for fetal indications [4]. Even though not provided on maternal request, the wide range of approved indications ensured Finnish women easier access to abortion, especially before 12 gestational weeks. Following revision of the abortion law in 2022, termination of pregnancy can now be obtained upon request until the 12^th^ gestational week [4, 5]. However, approval from a national board (National Supervisory Authority for Welfare and Health) is still needed between the 12^th^ and 24^th^ gestational week. This national board comprises a civil servant and three members with judicial, medical, and social expertise [4, 5].

In Iceland, the first law on induced abortion came into effect in 1935 [6]. With some modifications in 1938, the law described strict indications akin to those later used in other Nordic countries, but also allowed for consideration of the woman’s health and her social situation. The way the law was administered allowed for gradual liberalization in the 1970s. A new law in 1975 opened for approval of pregnancy termination on a wide range of social indications, but not on maternal request. However, liberal social indications allowed a gradual change towards respecting women’s wishes. A two-person approval (two physicians or a social worker and physician) was required. A three-person national committee (physician, social worker, lawyer) had to approve termination after the 12^th^ gestational week (> 11^+6^ weeks) based on specified medical and social conditions. While the 12^th^ week up to 16 weeks was a “grey zone” where two physicians could approve a request for termination, the national committee dealt with applications from 16^+0^ to 21^+6^ weeks. In 2019, the Icelandic parliament passed a new law allowing abortion on request up to 21^+6^ gestational weeks [6].

As early as 1915 pro-choice activists in Norway proclaimed women’s rights to abortion on request based on legislature on inheritance rights for children born out of wedlock [7]. In 1934 the Ministry of Justice appointed a committee to work on new abortion legislation. The work was never completed due to political opposition. Pregnancy termination remained in the penal code until 1960, when the Norwegian parliament passed an abortion law based on strict indications like the requirements in Denmark and Sweden. Approval had to be obtained with an application signed by two physicians. The law did not come into effect until 1964. During the following years applications were increasingly approved, reaching 94% in 1974. In 1975, a new abortion law was ratified including a broader range of social indications. As of January 1979, terminations were allowed on women’s request up to the 12^th^ gestational week. Hospital-based abortion commissions approved applications from the 12^th^ and up to 21^+6^ gestational weeks on conditions outlined in the law [7].

The Swedish parliament passed the first law on elective abortion in 1938 [8]. It granted pregnancy termination only on a few strict medical indications like the Danish law. From 1946 the Swedish law allowed terminations on broader social indications. During the 1960s, a more liberal change in societal attitudes concerning sexuality and abortion prevailed to a larger extent in Sweden than in the other Nordic countries. This led to an increased approval rate for abortion applications and paved the way for legal changes in 1974, giving Swedish women the right to abortion on request until the 18^th^ gestational week. At later gestational weeks, abortions can still only be performed after an application is deemed justifiable, provided the fetus is not considered viable by the National Board of Health and Welfare’s (NBHW) special committee (Rättsliga Rådet). Professionals represented in this committee are a gynecologist, social work counsellor, psychiatrist, a judge, a member of the national parliament and a representative from the NBHW. In case of a need for pregnancy termination to save the woman’s life no application is needed [8].

The revised abortion laws in the Nordic countries during the 1970s also provide guidance and directions for abortion surveillance, counselling on pregnancy, as well as sexual and contraceptive matters, and specified which health professions were to be responsible for the abortion and contraceptive services provided.

In this study we assessed trends for induced abortion epidemiology across the Nordic countries considering the legal similarities and diversities. We also discuss these trends in the light of new medical innovations and changes in practical and legal provisions that may have had an impact on these trends.

## Materials and methods

The new abortion legislations enforced case-based surveillance in all Nordic countries. In the following we describe the different national abortion registers.

The Danish abortion register was set up in 1973, when the law on abortion on request came into effect [9, 10]. The register holds retrospectively data from 1970. Paper-based individual case report forms (CRFs) were used through 1994. Thereafter information was collected through the national patient register (Landspatientregisteret). From 2004, when private clinics and privately practicing gynecologists were allowed to perform abortions, information from reporting systems held at Statens Serum Institute (covering data for reimbursement for health services in the private sector) were added to the abortion register [9, 10].

In Finland individualized data on abortions have been collected since 1950 [11]. The abortion law obliged physicians to report cases to the abortion register maintained by the Finnish Institute for Health and Welfare within four weeks of the procedure. The data were computerized in 1977. Currently computerized data are available from 1983 onwards. From that time a standardized paper-based CRF contained information on the woman’s social and reproductive background, the reason(s) for the request, the procedure used and any early complications.

Before 1975 the Directorate of Health in Iceland gathered information through abortion applications [12]. Changes in the abortion law from 1975 introduced a standardized CRF comprising social and demographic variables, pregnancy history (in summary), reasons for applying, legal aspect of approvals and method of termination [12]. From 1984 the data were electronically reported. When Iceland changed the abortion law in 2019, the data collection system changed to direct retrieval through the hospital discharge register and the register of primary health care contacts [12].

From 1940 hospitals in Norway forwarded abortion applications to the county health office, which conveyed summarized data to the Norwegian Directorate of Health (Helsedirektoratet) [7]. The abortion law from 1975 authorized Statistics Norway as the receiver for a new mandatory CRF. Since 2006 the Norwegian Institute of Public Health has administered the Norwegian abortion register, including data from 1979 [13]. From 2017 data are reported solely electronically [13].

Statistical information on induced abortion stretches back to 1955 in Sweden [8]. The data collection became more comprehensive after 1974. Up to 1995 the data were collected by Statistics Sweden and published by the NBHW. Since then, clinics and hospitals have reported directly to NBHW. The data are collected anonymously on summary sheets where one line represents one woman/one abortion covering categorized variables on maternal age, gestational age, previous abortion(s), method of abortion and geographical region (county) [14]. Data are available on-line from 1983 onwards [14].

In the present study we have summarized data from the national registers and what has been published on the Nordic websites for induced abortions [15]. Furthermore, we have undertaken new analyses from these sources.

Because the study populations are complete national populations, we have not performed other statistical analyses as there is no sampling of study participants. We present the data in rates per 1000 women aged 15-49/year, and in proportions where the denominator is defined in the presented figures.

### Ethics statement

This study displayed data already published on a Nordic website [15], from Websites belonging to the different abortion registers (Denmark [9, 10], Finland [11], Iceland [12], Norway [13] and Sweden (data repository) [14]). As the data were already in the public domain and anonymous, no Institutional Review Board approval was required. This was confirmed by the Institutional Review Board, University of Oslo, Norway (REK Regional Committee for Medical Research Ethics Southern Norway, Section C, reference rt.uio.no #6181108). For the same reasons, additional analysis within the abortion registers in Iceland, Norway, and Finland, do not need institutional approvals as the analyses are done by personnel within the registers and only summary sheets of data, aggregated in 5-year age-groups, were shared with the study team as part of the registers service to researchers and other institutional bodies.

## Results

**Fig 1:**
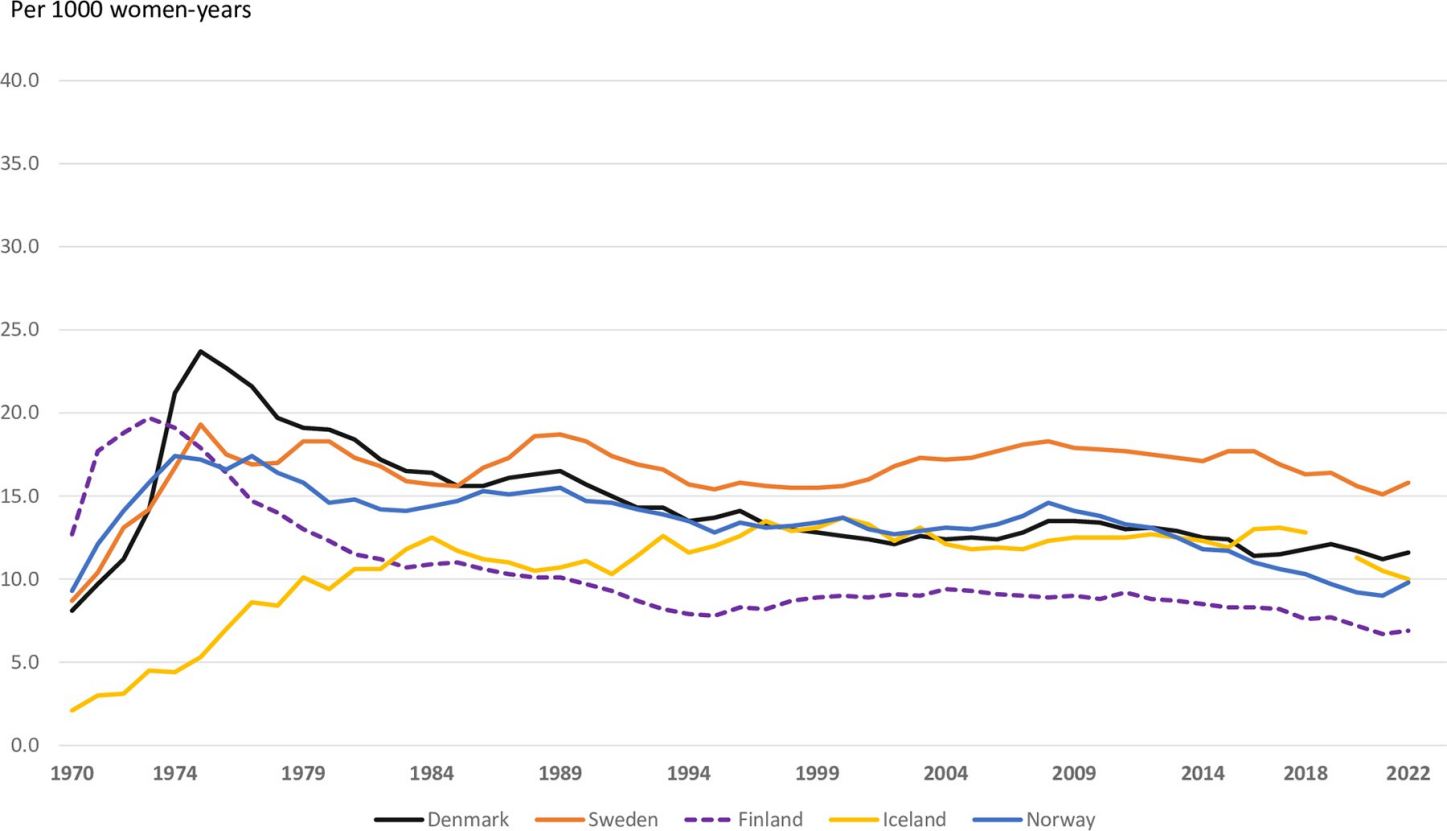
Induced abortions per 1000 women 15–49 years, Nordic countries, 1970–2022.

The numbers of induced abortions across the Nordic countries reflect the yearly size of the female population at reproductive age in each country. The total number of induced abortions has decreased from approximately 100 000 in 1975 (ranging from 274 in Iceland, 15 132 Norway, 21 547 Finland, 27 884 Denmark, to 35 526 in Sweden) to a total of 71 000 in 2022 (897 in Iceland, 11 967 Norway, 7 935 Finland, 14 660 Denmark, and 35 450 in Sweden). The abortion rate increased in all countries during the early 1970s and continued to increase in Iceland to the early 1980s (Fig 1). For Iceland the abortion rate has been stable since 1984 (11-12/1000 women/year). In Finland a remarkable decrease in abortion rates was observed from the 1980s to the mid-1990s, while a steadier decrease was seen in Norway and Denmark. Sweden has had the highest abortion rates among the Nordic countries since the late 1980s.

**Fig 2:**
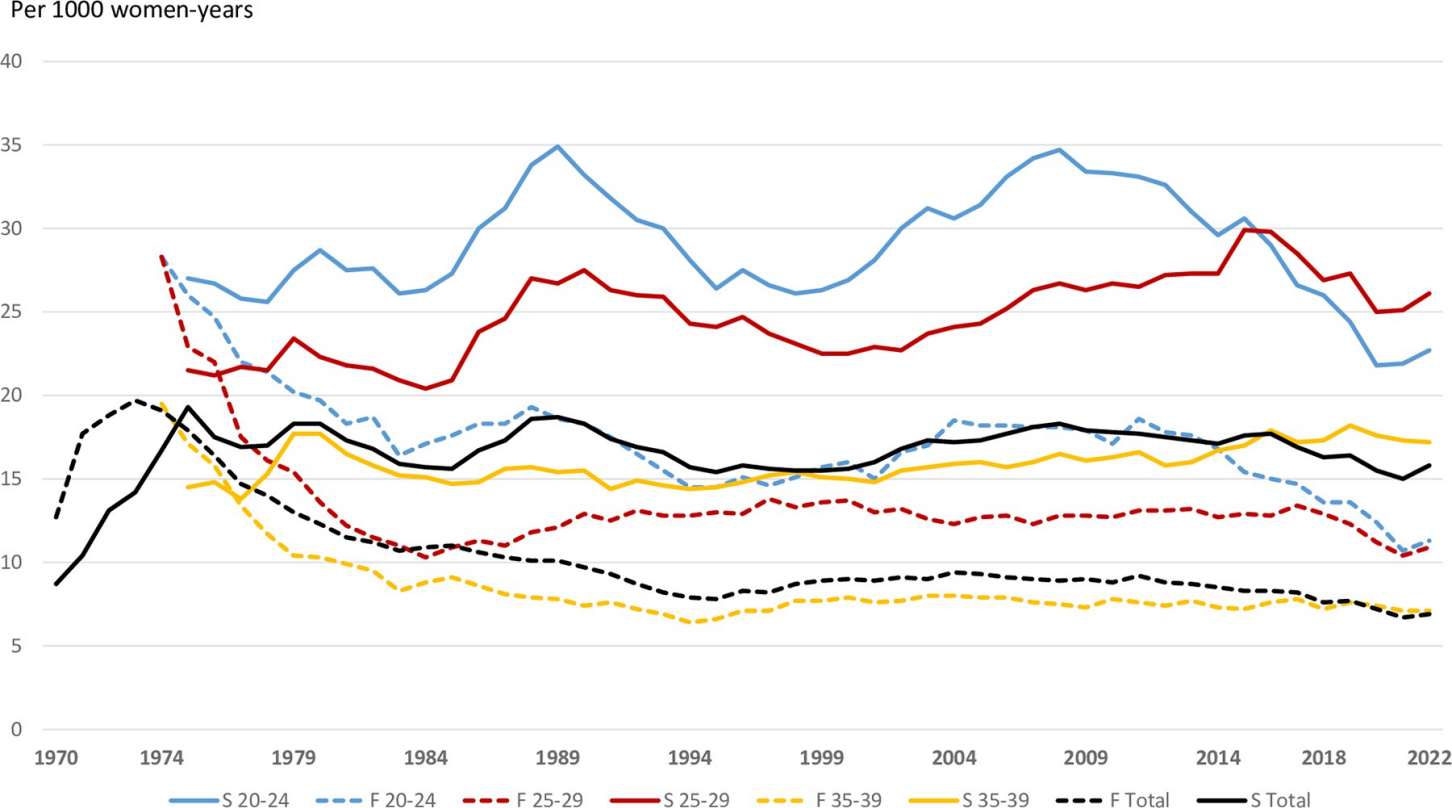
Selected age-specific induced abortion rates per 1000 women/year, Sweden (bold lines) and Finland (dotted lines), 1970–2022.

Age-specific abortion rates followed the same patterns as the overall abortion rates. Sweden has had the highest and Finland the lowest 5-year age-specific abortion rates. In each 5-year age group abortion rates in Sweden have been twice as high as in Finland (Fig 2), while the general and age-specific rates for Denmark, Iceland, and Norway have been in between.

**Fig 3:**
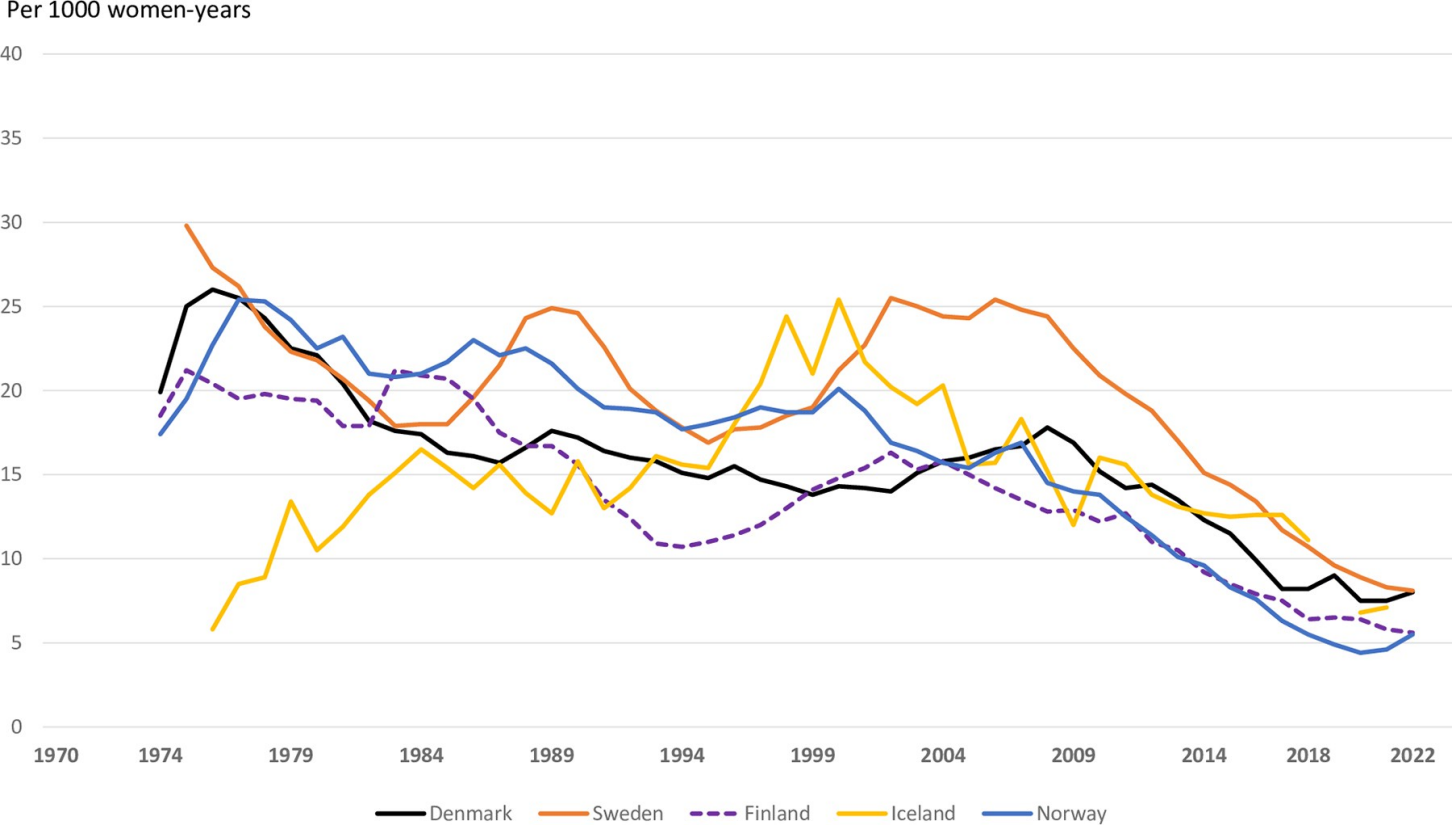
Induced abortion rates per 1000 women ≤ 19 years by Nordic country, 1974–2022.

For women 19 years or younger abortion rates decreased in all countries after the year 2000 (Fig 3). The age-group 20–24 years has had the highest abortion rates in all Nordic countries, but over the last years these rates decreased steeply. Currently the age group 25–29 years have had the highest abortion rates in Sweden, Norway, and Iceland, with no difference between the age groups 20–24 and 25–29 years in Finland and Denmark.

**Fig 4:**
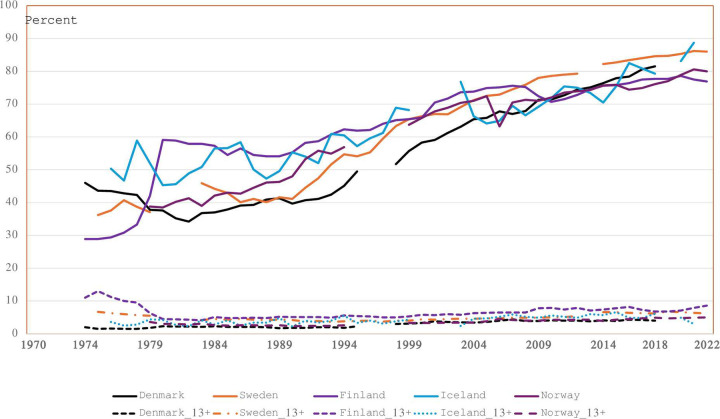
Gestational age ≤ 8 weeks (upper panel) and ≥ 13 weeks (lower panel) at the time of termination, Nordic countries, 1974–2022.

Over the years increasing proportions of induced abortions have been performed at relatively early gestational lengths in all Nordic countries. In Finland the proportion of induced abortions before the 9^th^ gestational week nearly doubled from 1978 to 1980 when there were changes in the upper gestational length limit (Fig 4). For Denmark, Norway, and Iceland there was a gradual increase of early 1^st^ trimester terminations from the early 1980s, while the same trend started at the end of the 1980s in Sweden and Finland. In 2022, 86%, 86%, 80% and 77% of all induced abortions in Sweden, Iceland, Norway, and Finland, respectively, were performed before the 9^th^ gestational week.

For 2^nd^ trimester induced abortions the proportion has remained low despite country-specific legislation differences. For Finland, the proportion of 2^nd^ trimester terminations decreased by more than 50%, from 10–11% before 1979, to 4–5% after 1979. Since 2000, the proportion of 2^nd^ trimester abortions increased again to 7–8% in Finland. For the other countries, except Sweden, the proportion of 2^nd^ trimester procedures comprised 4–5% until the mid-1990s and increased thereafter to 6% in recent years.

**Fig 5:**
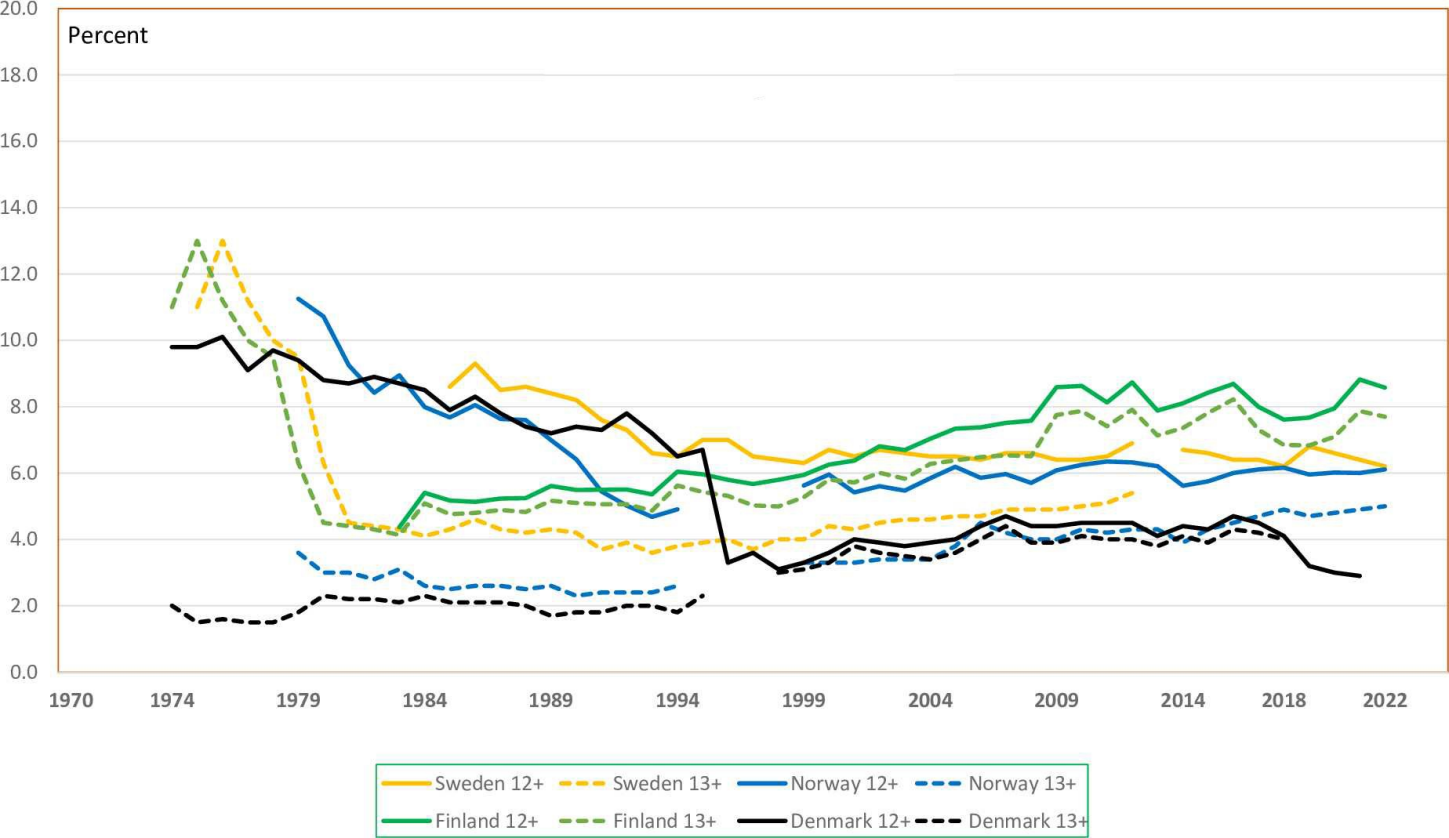
Gestational age ≥ 12 and ≥ 13 weeks at the time for termination, Sweden, Norway, Finland, and Denmark, 1974–2022.

Fig 5 displays the proportion of 2^nd^ trimester abortions defined as ≥12 or ≥ 13 gestational weeks for Denmark, Norway, Sweden, and Finland. The gap represents abortions during the 12^th^ gestational week. This gap has been steady for Finland, but has been larger, though decreasing, for Norway and Sweden. The largest gap was seen in Denmark. This gap nearly closed when Denmark changed the surveillance system to electronic retrieval of data in 1995.

More detailed information on 2^nd^ trimester abortions has been available from Finland, Norway, and Iceland. The proportion of early 2^nd^ trimester (13–15 gestational weeks) to later 2^nd^ trimester abortions (16–22 gestational weeks) remained relatively constant at around 50% since the early 1980s in Finland, while this proportion increased from under 40% in the mid-1980s to nearly 70% over the recent years in Iceland. In Norway, an opposite trend with increasing later 2^nd^ trimester abortions from 30% in the early 1980s to nearly 60% over the last years was noted.

**Fig 6:**
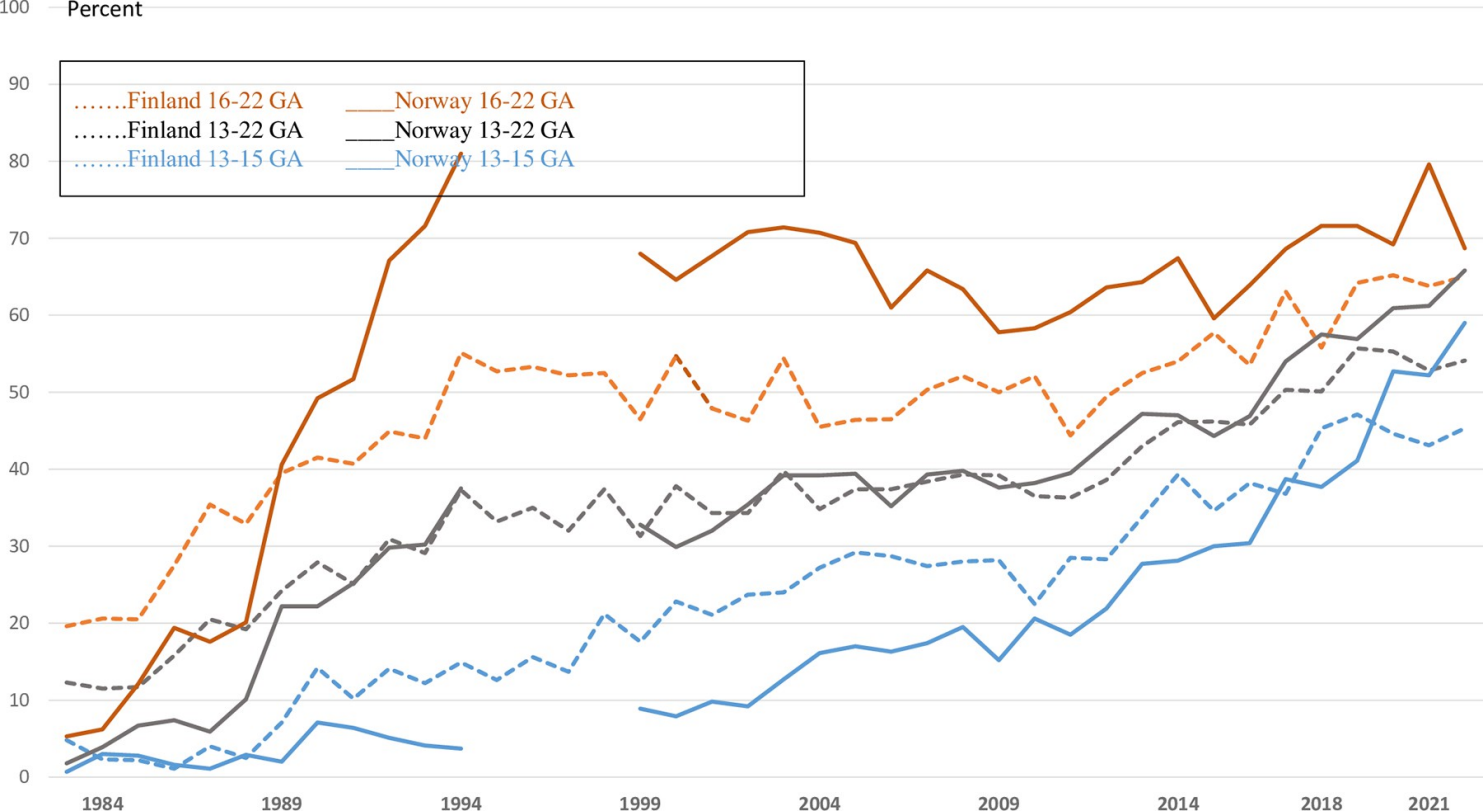
Proportion of 2^nd^ trimester induced abortions on fetal indications by gestational ages 13–15 weeks/16-22 weeks, Finland, and Norway.

A trend for increasing proportions of procedures on fetal indications was seen from the mid-1980s to the mid-1990s. This trend rose again after 2010, reaching 54% and 66% of all second trimester abortions in 2022 in both Finland and Norway (Fig 6). The proportion of late compared to early 2^nd^ trimester abortions on fetal indications has over the years been higher for late (Fig 6) compared to early 2^nd^ trimester abortions (Fig 6), reaching 64–65% and 70–72% in the late group in 2019–2021 in Finland and Norway, respectively. Iceland has had a higher proportion of abortions due to fetal indications than Norway and Finland, with large year-to-year variations due to small numbers of 2^nd^ trimester abortions (data not shown), ranging between 80–90% of all late 2^nd^ trimester abortions after 1990.

**Fig 7:**
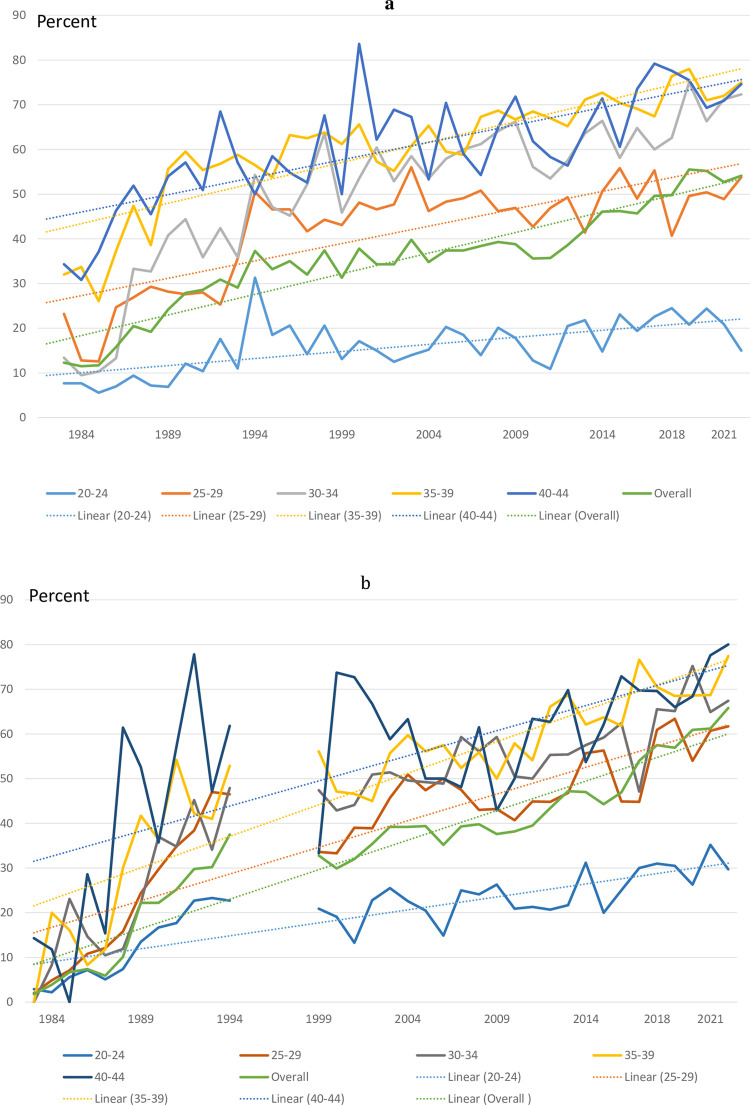
a. Fetal indication for gestational age > 12 weeks to all 2^nd^ trimester induced abortions by maternal age, Finland, 1983–2022 (dotted lines = linear trend). b: Fetal indication for gestational age > 12 weeks to all 2^nd^ trimester induced abortions by maternal age, Norway, 1983–2022 (dotted lines = linear trend).

Fetal indications increased from 1984 for the 5-year age groups over 30 years age reaching above ≥70% of all 2^nd^ trimester abortions in Finland in 2022 (Fig 7A). Trend lines indicate a steady increase in proportions of 2^nd^ trimester abortions on fetal indications in all age groups (Fig 7A). A similar age specific pattern was found for Norway (Fig 7B) with larger year-to-year variations due to smaller numbers of 2^nd^ trimester abortions than observed in Finland.

The proportion of very late 2^nd^ trimester abortions (≥19^+0^ gestational weeks) increased over the study years reaching 1.4% and 1.8% in Norway and Finland, respectively, in 2021. Over the last years this proportion of very late to all 2^nd^ trimester abortions remained higher in Norway (33–36%) than in Finland (20–22%). In these analyses Swedish data resembled the Finnish data considering that the definition of very late 2^nd^ trimester abortions in Sweden was reported as of >18^+0^ weeks.

## Discussion

All Nordic countries changed their abortion legislation during the 1970s and women gained liberalized access to abortion. The general abortion rates rose subsequently in all Nordic countries during the 1970s as a real increase, but partly because of more complete surveillance. None of the countries had reliable data on illegal abortions before the liberalization. The peak turned around 1976–78 in all countries, except for Iceland where the rate increased up to the mid-1980s (Fig 1). In Denmark and Finland, the general abortion rates then declined during the 1980s. In the other Nordic countries rates varied through the years around the millennium shift. From 2010 there has, however, been a moderate decline of the general abortion rates in all countries. The country-specific differences remained constant after the mid-1980s and are lower than reported for Western Europe and North America over the years 2015–19 [16].

As the abortion laws have been liberal and granted pregnancy termination in nearly any case within a reasonable gestational length, there has been minimal need for Nordic women to seek abortion abroad. A few women from outside the Nordic countries travel to our countries for abortion services (for example Polish women where abortion is illegal [17]).

Finland has had the lowest abortion rate in Europe among countries with reliable statistics. In the Netherlands abortion rates have varied between 7-8/1000 women for the age group 15–49 years after 2010 [18], which is lower than in Norway, Denmark, Iceland, and Sweden. While rates decreased in the Nordic countries in recent years, the general abortion rates increased in England and Wales, reaching 18.6/1000 women aged 15–49 years in 2021 [19], which is higher than in Sweden and the US [20]. In 2020–21 the general abortion rate in Sweden remained 25–30% below the highest abortion rates in the world [21].

The age-specific rates have reflected the general rates (Fig 2). From the early 1980s age-specific differences as seen in data from Sweden and Finland were also noted in the other Nordic countries. The pattern with the 25–29-year groups having a relatively higher rate than the 20–24 age-groups has also been reported from the US [20] and New Zealand [22]. Teenage abortion rates have more than halved in all Nordic countries since the millennium shift. Similar trends have been observed in England and Wales [19], US [20, 23] and New Zealand [21]. This was not compensated by higher teenage birth rates in any Nordic country [24], nor in England and Wales [19], the US [20] or New Zealand [22].

There has been a remarkable shift towards earlier terminations before the 9^th^ gestational week starting in the mid-1980s in Denmark and Norway, and a few years later in the other Nordic countries (Fig 4). Whereas around 40–50% of terminations were done before the 9^th^ gestational week in earlier decades, this proportion has reached 80–86% in recent years. This indicates a shift towards an increasing awareness on the need for abortion decisions at an earlier gestational age among women. Availability of medical abortion has facilitated this process.

The first randomized clinical trials on the benefits of routine ultrasound screening in the 2^nd^ trimester were published in 1984 (Norway) [25, 26], in 1988 (Sweden) [27] and in 1990 (Finland) [28]. By the early 1990s ultrasound screening was well established in all Nordic countries [27–30]. During the implementation period the proportion of 2^nd^ trimester abortions remained stable in all Nordic countries (Fig 4; Denmark ~2%, Finland ~5%, Sweden 3–4%, Iceland 2–4%, Norway ~3%). In actual numbers, between 560 and 660 2^nd^ trimester abortions were performed during 1985–1994 in Finland, and they have remained around 600. In Norway the corresponding actual numbers have ranged from between 350 and 400 during the implementation period to around 500 in recent years. In both Finland and Norway, the number of late 2^nd^ trimester abortions on maternal/social indications decreased at the same time as late 2^nd^ trimester abortions on fetal indications increased (Fig 6). This shift in maternal/social indications was in line with the observation that women sought pregnancy terminations gradually at earlier gestational ages. Over recent years the proportion of late 2^nd^ trimester abortions has increased both in Finland and Norway, but more because of a lower total number of abortions than because of an increase in actual numbers of late 2^nd^ trimester abortions. The proportion of early 2^nd^ trimester abortions on fetal indications increased, however, from the early 1990s in both Finland and Norway and reached 50% of all abortions at these gestational ages, while the remaining proportion procedures were performed on maternal/social indications. This increase may also be attributed to improved screening methods, especially among older expectant mothers.

Denmark has offered combined first trimester screening along with second trimester ultrasound examination for fetal anomalies from 2004 [30, 31]. For Down’s syndrome this led to increased detection rates, which amounted to additional 30–40 early 2^nd^ trimester terminations every year [31, 32]. Early ultrasound screening started 1999 in Iceland with combined first trimester screening being established in 2004, while expanded fetal screening programs were introduced in Finland in 2007.

Over the years routine ultrasound examination among women requesting abortion has become more common and in places it has been mandatory to detect non-viable pregnancies that would thus not have to undergo “termination”. Routine ultrasound for women seeking termination of pregnancy may have had a minor effect towards lowering rates, but this has not been investigated.

Pregnancies terminated during the 12^th^ gestational week has been in a “grey” zone, except in Sweden, where abortion on demand was accessible up to 18^+0^ week. In 1979 Finland lowered the upper limit from the 16^th^ to the 12^th^ gestational week. Already by 1983, the proportion of terminations in the 12^th^ gestational week was small (Fig 5), and this gap, though increasing, has remained small. In Norway and Denmark terminations during the 12^th^ week was like in Sweden. In Norway and Denmark terminations in the 12^th^ week were registered as 1^st^ trimester abortions. Recently differences in terminations at ≥ 12 gestational weeks have been minimal between Sweden and Norway, while Finland has a higher proportion of 12^th^ week terminations. In Denmark this gap lessened after 1995 when Denmark shifted from CRF registration to retrieving data from the patient administrative systems. It is likely that the law changes in Finland from 1979 had an impact on how gestational length was set. In Norway and Denmark, it had had had juridical implications how gestational age was determined, but not in Sweden with its 18^th^ week limit. The increase (1–2%) observed in all countries over recent years in the proportion of 2^nd^ trimester abortions defined as ≥12 gestational weeks is thus a consequence of lower numbers of 1^st^ trimester abortions, improved ultrasound dating of gestational length and of national health authorities stressing that gestational length should be securely validated in the transition zone between 1^st^ and 2^nd^ trimesters (Finland from 1979, Sweden from 2013).

After 2019 Iceland has had the most liberal abortion law among the Nordic countries allowing induced abortion by request up to 21^+6^ weeks. This has not increased the number of very late nor 2^nd^ trimester abortions overall.

As Finland and Norway have had systems for surveillance of induced abortions and for in- and outpatient services in hospitals, assessment of completeness and quality of reporting has been possible. The Finnish register was compared to the hospital discharge register at 10 hospitals, over three randomly selected months in 2011, comprising 43% of all induced abortions at that time [33]. The coverage of data on abortion procedures from the in- and outpatient registers was 97% in the abortion register. More detailed comparisons of different variables (timing of abortion, indications, procedures) showed good validity [33]. Data from 2008 to 2015 from the Norwegian abortion register was validated through procedure and international classification of diseases (ICD-10) codes for induced abortion in the Norwegian Patient Register [34]. For each year more abortions were registered in the abortion register than shown by data from the patient register. There was systematic “overreporting” of surgical abortions and “underreporting” of medical abortions in the patient register compared with the abortion register. The two registers have different templates for identification of cases and use different coding systems which may explain part of the differences. Denmark, Sweden, and Iceland (as of 2019) generate the abortion statistics through data from the patient administrative systems, and thus have limited access to variables and possibilities for quality assurance of what is registered. Finland has decided to continue detailed reporting of all induced abortions to the national abortion register with the new legislation since September 1^st^, 2023, while Iceland decided to close the CRF based abortion register in 2019. It is a political decision how much information societies need to accrue about induced abortion.

The changes in the abortion laws during the 1970 strengthened and introduced structured CRFs on mandatory surveillance from hospitals performing abortions to national registers. As the age-specific and overall trends of induced abortion have been alike across the Nordic countries across so many years, we find it unlikely that underreporting or misclassification of induced abortion to spontaneous abortion have been a limitation for the generalizability of our study.

Awareness and use of contraception is high in the Nordic countries [34]. During the late 1980s and 1990s effective use of hormonal contraception prevented unplanned pregnancies that went on to births. The Nordic countries have managed to keep the number of teenage births low at the same time as induced abortions decreased among younger women (Fig 3) [24, 35, 36]. To illustrate the magnitude of these changes among women under 20 years over the recent years, the induced abortion rate has been lower than estimated contraceptive failure rates if all teenagers had been sexually active and used a copper intrauterine contraceptive device [37, 38]. The right to safe induced abortion is challenged in the US and several other high-income countries [39]. The Nordic countries have taken a more liberal position for a long time and are heading towards more rights for women to determine themselves whether to continue pregnancy, even at 2^nd^ trimester gestational lengths (Sweden 18^+0^, Iceland 21^+6^, law proposals Norway 17^+6^ and Denmark 18^0^ GA). As “birth” and viability are generally acknowledged to apply at least by 22 weeks, extension of limits beyond that time is unlikely in the Nordic countries.

Liberalization of the abortion laws in the Nordic countries during the early 1970s took place at the same time as women’s rights to higher education, gender equality, sexual and reproductive rights increased. With the liberalization of the abortion laws followed widespread strengthening of sexual education, especially in schools. This was allied to improved access for increasingly subsidized or low-cost contraception in our countries. Over the years openness on sexual and reproductive rights has evolved in the Nordic countries where easy (public) access to affordable high quality health services on reproductive issues has become mandatory.

## Conclusion

Fifty years with liberalized abortion laws has given Nordic women rights to safe high quality abortion services. After an increase in abortion rates during the first years of liberalization, the general abortion rates stabilized and even decreased to a proportionally lower level than seen half a century ago. More recently there has been a remarkable decrease of abortions among women under 25 years age without higher concomitant birth rates. In all countries women now decide on abortion at an earlier gestational age, mostly terminated before the 9^th^ gestational week by medical rather than surgical means. Introduction of 2^nd^ trimester ultrasound screening during the late 1980s increased the number of 2^nd^ trimester abortions on fetal indications with no overall increase in the proportion of 2^nd^ relative 1^st^ trimester abortions. Refinements of ultrasound screening and non-invasive prenatal diagnostic methods have led to a slight annual increase in early 2^nd^ trimester abortions from 1990 and onwards, while late 2^nd^ trimester abortion leveled off in the early 1990s and increased again after year 2010. Country-specific differences have remained over the 50 years of observation, indicating that there are some cultural differences in relation to abortion epidemiology across the Nordic countries, which in so many other respects share liberal and progressive cultural values.

## Supporting information

S1 Data (XLS)
